# Why is heartache associated with sadness? Sadness is represented by specific physical pain through verbal knowledge

**DOI:** 10.1371/journal.pone.0216331

**Published:** 2019-05-01

**Authors:** Mariko Shirai, Takahiro Soshi

**Affiliations:** 1 Department of Psychology, Doshisha University, Kyotanabe, JAPAN; 2 Graduate School of Frontiers Biosciences, Osaka University, Suita, JAPAN; University of Rome, ITALY

## Abstract

People describe sadness as “heartache.” The link between sadness and physical pain such as heartache has been empirically proven; however, the mental foundations that support the connection between sadness and pain remain unclear. The present study hypothesized that the connection between sadness and specific physical pain is established by concepts referred to as “sadness-pain concept,” which are internalized based on features relating to interactions between the body and external situations. We examined the use of pain-related onomatopoeias as metaphorical words for expressing sadness, setting three primary goals for our study: (i) to identify sadness-pain words usable for both sadness and physical pains, (ii) to determine the specific sadness-pain words used for certain sadness situations, and (iii) to reveal the pain-related characteristics that are linked to such sadness situations. Sixty-nine participants were asked to rate 28 pain-onomatopoeic words in terms of the types of emotions, sadness situations, body parts, and characteristics of pain respectively. Consequently, seven words were identified as sadness-pain words. Furthermore, the specific sadness situations related to each sadness-pain word were determined. Situation-dependent sadness-pain words, for example, *zukin zukin* for loss, were found to be associated with specific body parts and pain properties. These findings indicate that the shared representation of sadness and physical pain as an emotional concept is based on interactions between the body and external situations.

## Introduction

A common phrase used to express the psychological state of sadness is “my heart is broken,” referring to physical pain, which is defined as an unpleasant sensory and emotional experience associated with actual or potential tissue damage, or described in terms of such damage (The International Association for the Study of Pain: IASP). Such an expression has metaphoric significance because those who utter it are not actually experiencing physical pain evoked by invasive stimuli. Although the close connection between the psychological state of sadness and specific physical state of pain has been verified through our daily life, the mental foundations that support the connection between sadness and physical pain remain unclear. This unresolved question may relate to the traditional mind-body problem as considered by René Descartes (1596–1650) [1], that a physical state is translated into a psychological state through an expression of physical pain.

The mind-body linkage could be instantiated by the “sadness-pain concept,” which can be used to represent both sadness and physical pains. Such emotional concepts are composed of a set of features [2], which function as a unified concept in not only distinguishing major emotional concepts such as sadness and happiness, but also discriminating between subtypes within the same major emotion (e.g. [3]). Such feature-based representation of emotional concepts is fundamental for constructing “knowledge of emotion” as a collection of emotional concepts, which enables us to identify our own and others’ emotional states in daily situations [3, 4]. Sadness-pain concept, therefore, is assumed to possess overlapping features, which probably makes it possible to express sadness as well as physical pains.

Two key elements may be related to overlapping features underlying the sadness-pain concept. The first element is the body, as physical signals play an important function in both sadness and pain [5]. Among various related features, such as external triggering events and subjective or psychophysiological responses [6–9], bodily expressions construct the emotions [10, 11], as per William James’s viewpoint of the association between emotion and bodily response [12]. In fact, the behavioral expression “heartache” is frequently utilized as a sadness-expressive word in describing loss of loved one or social rejection [13–16]. Based on autonomic physical-response properties, sadness can be divided into subtypes, such as loss and failure. Though both loss- and failure-sadness increase the skin conductance responses, for loss-sadness, it takes longer for the levels to return to normal than that for failure-sadness [16]. Loss- and failure-sadness are also conceptualized differently based on their physical-activation properties [3]: loss-sadness is associated with less-overt physical actions, which are performed to cope with psychological damage [17, 18]; on the other hand, failure-sadness is associated with overt behaviors, which are performed to achieve goals [19, 20]. Physical pain in specific body parts is communicated to the brain through nociception [21, 22], and this motivates organisms to avoid the triggering threat and move to safety [14]. In order to determine subjective criterion regarding pain, the relationship between body parts and pain properties is essential, as this can help individuals to learn and recognize specific feelings of pain (e.g., a stomach pain) [23].

The second element is external situations surrounding our physical entity. Through the course of their lives, people are exposed to various situations and experience a range of emotional and mental states [24, 25]. In particular, sadness can occur in response to social events such as social rejection, homesickness, or loss (e.g., loss of loved one) [26], which occur during interactions of the body with the social environment. Actually, sadness comprising loss- and failure-sadness [15, 16] is represented differently in verbal concepts based on their physical-activation features [3], and can induce different autonomic physiological responses [16]. Thus, sadness possesses properties of interaction between the social situations and body. Physical pain is also inseparable from external situations. We, as signal-enabling organisms, can interpret painful stimuli as an alarm signal and consequently take physical action to avoid life-threatening situations [27]. Once adaptive natures of the pain signal [27, 28] become dysfunctional, human physical behaviors may neither maintain homeostasis nor take appropriate actions in correspondence to situational demands. Chronic pain, for example, affects adaptive behaviors [29]; people with chronic pain can develop impaired behavioral inhibition, as they must change their perceptions of pain as a result of the situational demands they experience [30]. Taken together, the two key elements of situation and body are not divisible and interact with each other to construct sadness-pain concepts.

To examine sadness-pain concepts internalized in the mind, one useful method is to test verbal knowledge, because verbal items such as words encode internalized emotional concepts [4, 31, 32]. The Japanese language has numerous words or onomatopoeias for expressing not only emotional, but also painful states. Onomatopoeias are defined as words that sound similar to those they represent and include sounds associated with nature, animals, and humans [33] (e.g. dog’s barking such as “*wan wan*” in Japanese and “*Bow wow*” in English); such terms can also represent various symbolic expressions that describe actions, behaviors, and human psychological states [33, 34]. Onomatopoeic expressions are acquired in early developmental stages [35, 36], and are used frequently in not only public (e.g. newspaper) and private (e.g. daily conversation) situations. They typically function as a modifier of predicates or nouns such as adverbs and adjectives and can vividly represent subtle differences and nuances of expressions [33]. For example, crying-related onomatopoeias can effectively represent different types of crying behaviors, such as overt (e.g., “*boro boro*” represents crying with large amounts of tears) and less-overt expressions (e.g., “*shiku shiku*” represents soft weeping) [3]. Pain-related onomatopoeias also selectively represent specific types of pains, distinguishing subtle differences in pain properties (Onomatope Lab: http://onomatopelabo.jp/index.html?medical); for example, some of these onomatopoeias are associated with headaches (e.g., “*gan gan*”: pain or noise echoing in the head), while others are associated with back pain (e.g., “*jin jin*”: incessant pain and numbness in the afflicted area).

The present study hypothesized that sadness and specific types of physical pain possess overlapping features that form a single verbal concept, which is topologically defined by physical properties and situations. To test this hypothesis, we selected pain-related onomatopoeias as possible elements of verbal concepts for expressing sadness, and performed three investigations: (i) identifying words that are applicable to pain and sadness, (ii) determining sadness-pain words that are appropriate to particular sadness situations, and (iii) revealing the kinds of physical characteristics (pain expressions and body parts) associated with sadness situations. Through these investigations, we sought to reveal psychological foundations for a shared mental representation between sadness and physical pain.

## Materials and methods

### Participants

Sixty-nine undergraduate students (36 male, 33 female) with a mean age of 20.39 years (*SD* = 1.25) participated in the study. All were native Japanese speakers and received course credit in return for their participation. Informed consent was obtained from all participants prior to the experiment. The experimental protocol was approved by the ethics committee of the faculty of psychology at Doshisha university and accorded with the 1975 Helsinki declaration and its later amendments.

### Experimental stimuli

The present study featured pain-expressive word stimuli, with their respective congruency with four factors being determined (pain-related: body parts, characteristics of pain expressions; emotion-related: types of emotions, sadness-related situations), as described below.

### Pain-expressive words

From an overall pool of 4,500 onomatopoeic and mimetic words, 28 words (e.g., *zuki zuki*: throbbing, grinding pain) were selected based on a priori categorization of pain-related words in the Japanese Onomatopoeia Dictionary [37] (see Table 1).

**Table 1:** The 28 pain-related onomatopoeias used in the present study.

	Stimulus words		Meaning
No.	Japanese	Transliteration	
1	しくしく	*shiku shiku*	Continuous spasms of dull pain
2	しくりしくり	*shikuri shikuri*	Repeated pricking pain
3	ずきずき	*zuki zuki*	Throbbing, grinding pain
4	ぐさぐさ	*gusa gusa*	Repeated stubbed with a sharp object
5	ちくちく	*chiku chiku*	Repeated pricking pain such as that induced by a needle
6	ずきんずきん	*zukin zukin*	Continuous throbbing pain
7	じんじん	*jin jin*	Continuous pain and numbness in the afflicted area
8	ひりひり	*hiri hiri*	An irritation in your throat or of your skin from physical injury or hot food
9	ぎしぎし	*gishi gishi*	Joint creaking pain
10	ちりちり	*chiri chiri*	A cut or rash that feels like it is burning
11	いがいが	*iga iga*	An itchiness or tickle in your throat
12	びりびり	*biri biri*	Incessant pain or irritation
13	がんがん	*gan gan*	Pain or noise echoing in your head
14	きりきり	*kiri kiri*	A stomachache with sharp pains
15	ずんずん	*zun zun*	Throbbing, grinding pain
16	むずむず	*muzu muzu*	Feeling itchy like you were bitten by bugs, or that bugs are crawling on you
17	ごりごり	*gori gori*	Itchiness causing pain
18	ごろごろ	*goro goro*	The pain of something in your eye
19	ぎんぎん	*gin gin*	Severe incessant headache
20	がじがじ	*gaji gaji*	Gnawing pain
21	ちかちか	*chika chika*	Flashing pain in your eyes
22	もぞもぞ	*mozo mozo*	Feeling itchy like you were bitten by bugs, or that bugs are crawling on you
23	うずうず	*uzu uzu*	Incessant dull pain
24	どきんどきん	*dokin dokin*	Violent throbbing
25	がくがく	*gaku gaku*	Trembling joints, such as knees
26	ぴりぴり	*piri piri*	Incessant pain or irritation
27	ぴきぴき	*piki piki*	Twitching and being on edge
28	いらいら	*ira ira*	An irritation in your throat or of your skin from physical injury or hot food

These explanations were sourced from “Japanese Onomatopoeia: The definitive guide” (www.tofugu.com/japanese/japanese-onomatopoeia/) and some words were translated based on the meaning of the Japanese dictionary.

### Body parts

Thirty-six physical parts of the body were selected for assessing the congruency between pain-expressive words and body parts, referring to the Anatomical Dictionary (https://anatomy1.net/?FrontPage#f0d042ac). These included not only outer physical areas, but also internal organs; specifically, the body parts were: head-neck (nine parts): head, face, eyes, nose, mouth, jaw, tooth, neck, and throat; trunk (five): shoulder, chest, abdomen, waist, and back; limbs (17): arm, elbow, hip, hand, leg, wrist, ankle, knee, shin, fingertip, tip of toe, heel, palm, dorsum of hand, sole of foot, dorsum of foot, and joints; and others (four): muscles, stomach, heart, and other internal organs.

### Characteristics of pain expression

Overt pain-expression properties were categorized into four types based on [38]: continuity (acute–chronic), activation (activation–deactivation), intensity (weak–strong), and acuity (sharp–dull). Pain-related studies have generally examined these characteristics in clarifying the properties of pain [39, 40]. Participants were asked to assess the characteristics of pain following the instruction: “To what extent does *zuki zuki* indicate activation (other pain properties)?”

### Types of emotions

Four basic emotions (sadness, happiness, anger, and fear) were selected based on the previous study [41]. Emotional feeling is occasionally represented by referring to somatic sensations; notably, as mentioned above, feelings of sadness are commonly represented by pain-related expressions such as “heart ache” [16].

### Sadness-related situations

Six sadness situations were chosen: loss, failure, breakup, loneliness, disease, and family rift; these were reported in [26] as situations that notably evoke sadness. These situations were specified through a cluster analysis and multi-dimensional scaling, with similarities identified among the situations.

### Procedure

First, the participants judged how suitable the pain-expressive words were for expressing the four major emotions (sadness, happiness, anger, and fear); responses were given using a 10-point Likert scale (“1” = “does not express,” “10” = “expresses very appropriately”). Thus, the participants rated a total of 112 pairs (28 words × four emotions). Second, they assessed how appropriately the pain-expressive words matched the six sadness-related situations. The congruency between the words and each situation was evaluated using 168 pairs (28 words × six situations), and also involved the use of a 10-point Likert scale (“1” = “does not express,” “10” = “expresses very appropriately”). Third, the participants rated the relationship between the words and the body parts (28 words × 36 body parts = 1,008 pairs), again using a 10-point Likert scale (“1” = “does not express,” “10” = “expresses very appropriately”). Finally, each word’s level of pain-expression was assessed (28 words × four pain-expressive properties = 112 pairs), based on a 10-point Likert scale (e.g., duration of pain: “1” = “short,” “10” = “long”).

The stimuli were presented randomly using Qualtrics survey software (http://www.qualtrics.com) and data were collected through online. For each pair, an instruction sentence regarding how the pain-expressive words should be assessed was positioned at the top of the screen and the 10-point rating scale was presented at the bottom. Participants reported their scores for each item by selecting on the appropriate area of the scale. Participants performed the tasks at their own pace, being given sufficient time to consider each question.

### Data analysis

**Fig 1:**
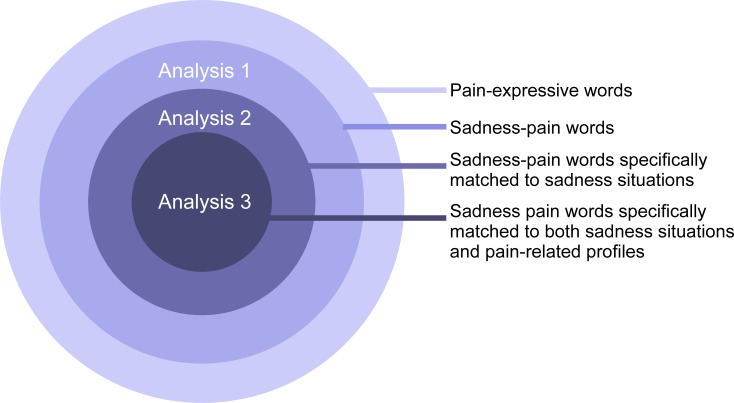
Analysis procedure. Analysis 1 aimed to identify the pain-expressive words that can also express the feeling of sadness. Analysis 2 aimed to identify the sadness situations that are specifically associated with the specified sadness-pain words. Analysis 3 sought to determine the kinds of pain-related profiles (body parts and characteristics of pain) that were represented by the situation-dependent sadness-pain words.

The analysis aim was to reveal how the relations between pain-expressive words and sadness emotion are instantiated into verbal expressions. Therefore, we examined (i) pain-expressive words that can also express sadness (analysis 1), (ii) the kinds of sadness situations that specifically match particular sadness-pain words (analysis 2), and (iii) the kinds of pain-related profiles (body parts and characteristics of pain expressions) represented by the sadness-pain words (analysis 3) (Fig 1).

In analysis 1, we specified the pain-expressive words that can suitably express sadness; that is, sadness-pain words. A hierarchical cluster analysis was performed based on the emotional-expressive scores (0–10) for each word in order to obtain word clusters with similar expressive patterns. Between-word distances were computed based on Euclidean distances, and the words were clustered using Ward’s linkage method. To specify clusters with a dominant sadness property, each word within the sadness cluster was analyzed using a one-way repeated-measures analysis of variance (ANOVA) with emotion (four levels: sadness, happiness, anger, fear) set as the independent variable. When the main effect was significant, post-hoc analyses using the Bonferroni method were performed to reveal whether the scores for sadness were significantly higher than those of the other emotions (*p* < .05). Greenhouse-Geisser correction of degree-of-freedom was also applied, if necessary.

In analysis 2, to identify the specific relation between each sadness-pain word specified in analysis 1 and the sadness situations, we conducted multiple-regression analyses for each word, using the congruency score with sadness as the dependent variable (y), the congruency scores with the sadness situations as the independent variable (x), and sex as the control variable. The regression analysis was conducted using the backward elimination method (the inclusion criterion was a coefficient (*β*) of *p* < .05 for the independent variable) and the significant model with the highest model-fitting value (adjusted *R*^*2*^) was adopted.

In analysis 3, we sought to determine the kinds of pain-related profiles that are linked to the sadness-pain words specified in analysis 2. Thirty-six body parts were initially clustered based on the congruity scores between the words and body parts, using Euclidean distances and Ward’s linkage method. Subsequently, multiple-regression analyses were conducted for each word to predict their respective congruency scores for the sadness situations, with pain profiles as the independent variable and sex as the control variable, and using the backward elimination method (*p* < .05). We consequently chose a significant model with significant *βs* and the highest model-fitting value (adjusted *R*^*2*^).

## Results

### Analysis 1: Sadness-expressive words

**Fig 2:**
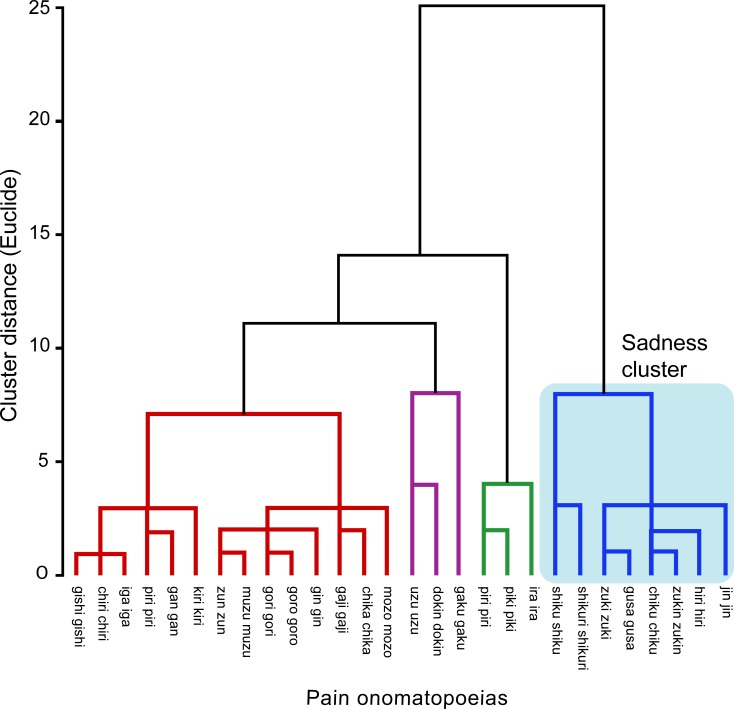
The 28 pain-onomatopoeias were grouped into four clusters based on similar emotion-expression patterns. The first cluster (blue line) consisted of the eight words with the highest congruent rating for sadness, which were referred to as sadness-pain words. The second cluster (red line) consisted of 14 words with relatively high congruency ratings for anger. The third cluster (purple line) was composed of three words that had high congruency with both happiness and fear. The fourth cluster (green line) comprised three words that were highly congruent with anger.

Hierarchical cluster analysis observed the four main clusters, which corresponded to the classification of the four major emotions (Fig 2); Table 2 shows the averaged congruency ratings between each emotion and the words in each cluster. Specifically, the first cluster comprised eight words that had a higher congruency rating for sadness (6.37 ± 1.45) than for the other emotions (happiness: 1.84 ± .57; anger: 3.47 ± .70; fear: 3.29 ± .41); these were consequently referred to as sadness-pain words (Fig 1). The second cluster comprised 14 words with relatively high congruency ratings for anger. The third cluster comprised three words that had high congruency ratings for both happiness and fear. Finally, the fourth cluster comprised three words that were highly congruent with anger.

**Table 2:** Congruency ratings between the 28 pain-related words and the four major emotions (*n* = 69).

	Stimulus words		Congruency ratings for emotions							
		Transliteration	Sadness		Happiness		Anger		Fear	
Cluster			Mean	*SD*	Mean	*SD*	Mean	*SD*	Mean	*SD*
1	*shiku shiku*	Continuous spasms of dull pain	9.55	1.39	1.42	1.23	2.55	2.29	3.26	2.57
	*shikuri shikuri*	Repeated pricking pain	7.28	3.02	1.58	1.61	2.10	1.71	2.41	1.99
	*zuki zuki*	Throbbing, grinding pain	6.46	2.92	1.64	1.59	3.91	2.70	3.86	2.66
	*gusa gusa*	Repeated stubbed with a sharp object	6.62	2.65	1.55	1.22	3.75	2.58	3.65	2.54
	*chiku chiku*	Repeated pricking pain such as that induced by a needle	5.58	2.95	1.77	1.45	4.19	2.64	3.16	2.53
	*zukin zukin*	Continuous throbbing pain	5.83	2.94	1.64	1.49	4.10	2.58	3.45	2.36
	*jin jin*	Continuous pain and numbness in the afflicted area	4.59	2.75	1.86	2.56	3.61	2.37	3.45	2.46
	*hiri hiri*	An irritation in your throat or of your skin from physical injury or hot food	5.04	2.88	3.30	1.79	3.55	2.42	3.10	2.28
2	*gishi gishi*	Joint creaking pain	2.90	2.36	1.81	1.55	3.71	2.60	3.12	2.37
	*chiri chiri*	A cut or rash that feels like it is burning	2.94	2.30	1.77	1.74	3.75	2.58	2.97	2.31
	*iga iga*	An itchiness or tickle in your throat	2.88	2.33	1.32	1.12	4.43	2.69	2.88	2.16
	*biri biri*	Incessant pain or irritation	2.84	2.34	2.84	2.51	4.29	2.81	3.70	2.61
	*gan gan*	Pain or noise echoing in your head	3.12	2.08	2.75	2.42	5.14	2.63	2.77	2.09
	*kiri kiri*	A stomach ache with sharp pains	4.23	2.65	1.48	1.15	5.10	2.84	4.22	2.64
	*zun zun*	Throbbing, grinding pain	2.70	2.23	3.04	2.54	3.06	2.38	2.45	2.05
	*muzu muzu*	Feeling itchy like you were bitten by bugs, or that bugs are crawling on you	2.43	1.81	3.88	2.78	3.12	2.00	2.84	2.14
	*gori gori*	Itchiness causing pain	2.30	2.16	2.59	2.59	4.12	2.64	2.68	2.32
	*goro goro*	The pain of something in your eye	2.16	1.62	3.01	2.56	3.74	2.91	2.17	2.01
	*gin gin*	Severe incessant headache	1.88	1.61	3.99	2.69	4.12	2.61	2.36	1.93
	*gaji gaji*	Gnawing pain	2.04	1.71	1.51	1.17	3.07	2.36	2.52	2.15
	*chika chika*	Flashing pain in your eyes	2.14	1.94	2.55	2.08	2.54	2.16	2.00	1.98
	*mozo mozo*	Feeling itchy like you were bitten by bugs, or that bugs are crawling on you	2.22	1.54	3.22	2.41	2.20	1.62	3.97	2.72
3	*uzu uzu*	Incessant dull pain	2.32	1.74	5.72	2.94	2.96	2.13	2.49	2.07
	*dokin dokin*	Violent throbbing	2.74	1.94	5.65	2.60	3.17	2.26	6.04	2.78
	*gaku gaku*	Trembling joints, such as knees	4.17	2.86	1.88	1.69	3.59	2.56	8.39	2.54
4	*piri piri*	Incessant pain or irritation	2.64	1.93	1.46	0.93	7.46	2.74	3.75	2.66
	*piki piki*	Twitching and being on edge	2.52	2.02	1.72	1.37	5.93	3.20	2.75	2.22
	*ira ira*	An irritation in your throat or of your skin from physical injury or hot food	2.45	2.11	1.17	0.70	9.22	2.04	2.54	2.18

To statistically test the dominant sadness property of the first cluster, each word’s congruency with the four emotions was analyzed, using an ANOVA with emotion set as the independent variable. The main effect of emotion was determined to be significant for all eight words (all *p*s < .01) [*shiku shiku*: *F* (3, 204) = 296.04, *ε* = .79, _*p*_*η*^2^ = .81; *shikuri shikuri*: *F* (3, 204) = 118.88, *ε* = .73, _*p*_*η*^2^ = .64; *zuki zuki*: *F* (3, 204) = 50.11, *ε* = .85, _*p*_*η*^2^ = .42; *gusa gusa*: *F* (3, 204) = 63.65, _*p*_*η*^2^ = .48; *chiku chiku*: *F* (3, 204) = 36.00, *ε* = .86, _*p*_*η*^2^ = .35; *zukin zukni*: *F* (3, 204) = 42.23, *ε* = .89, _*p*_*η*^2^ = .38; *hiri hiri*: *F* (3, 204) = 20.74, *ε* = .84, _*p*_*η*^2^ = .23; and *jin jin*: *F* (3, 204) = 11.44, *ε* = .83, _*p*_*η*^2^ = .14]. Post-hoc comparisons indicated that all of these words, except *hiri hiri*, had significantly stronger congruency with sadness than with the other emotions. Consequently, the first analysis identified seven sadness-pain words (*shiku shiku*, *shikuri shikuri*, *zuki zuki*, *gusa gusa*, *chiku chiku*, *zukin zukin*, and *jin jin*).

### Analysis 2: The relations between sadness-pain words and sadness situations

Multiple regression analysis for each word predicted the sadness congruency by the congruency for sadness situations and sex as a control variable (see Table 3). The regression model significantly predicted each word’s congruency with sadness (except *gusa gusa*) (all *p*s < .01) [*shikuri shikuri*: adjusted *R*^*2*^ = .33, *F* (2, 66) = 17.41; *zuki zuki*: adjusted *R*^*2*^ = .27; *F* (1, 67) = 25.88; *zukin zukin*: adjusted *R*^*2*^ = .25; *F* (1, 67) = 23.99; *shiku shiku*: adjusted *R*^*2*^ = .13; *F* (1, 67) = 10.97; *jin jin*: adjusted *R*^*2*^ = .20; *F* (1, 67) = 17.94; *chiku chiku*: adjusted *R*^*2*^ = .15; *F* (1, 67) = 13.41].

**Table 3:** Multiple-regression analysis results for the predicted congruency of the sadness-pain words regarding the sadness situations specified in Analysis 2.

	Sadness-pain words (Transliteration)						
Sadness situations	*shikuri shikuri* (Repeated pricking pain)	*zuki zuki* (Throbbing, grinding pain)	*zukin zukin* (Continuous throbbing pain)	*shiku shiku* (Continuous spasms of dull pain)	*jin jin* (Continuous pain and numbness in the afflicted area)	*chiku chiku* (Repeated pricking pain such as that induced by a needle)	*gusa gusa* (Repeated stubbed with a sharp object)
	*β*	*β*	*β*	*β*	*β*	*β*	*β*
Loss	.38*	.03	.51*	.20	−.02	−.07	-
Failure	−.04	.04	.06	−.06	−.11	.41*	-
Breakup	.17	.23	.16	.38*	.11	.17	-
Loneliness	.14	.53*	.18	.20	.46*	.17	-
Disease or injury	.31*	.12	.12	.08	.11	−.03	-
Family rift	.02	.14	.08	.00	−.05	.00	-
*adjusted R*^*2*^	.33*	.27*	.25*	.13*	.20*	.15*	-

Only significant weights are shown (*p < .05).

Specifically, *shikuri shikuri* was found to be congruent with sadness for both loss (*β* = .38, *t* = 3.40, *p* < .01, *VIF* = 1.26) and disease (*β* = .31, *t* = 2.73, *p* = .01, *VIF* = 1.26) situations. Further, *zuki zuki*’s congruency rating was significantly predicted by loneliness situations (*zuki zuki*: *β* = .53, *t* = 5.09, *p* < .01, *VIF* = 1.00), and that of *zukin zukin* was significantly predicted by loss situations (*zukin zukin*: *β* = .51, *t* = 4.90, *p* < .01, *VIF* = 1.00). *Shiku shiku*’s sadness-congruency score was significantly predicted by breakup situations (*β* = .38, *t* = 3.31, *p* < .01, *VIF* = 1.00), while that of *jin jin* was significantly predicted by loneliness (*β* = .46, *t* = 4.24, *p* < .01, *VIF* = 1.00) and that of *chiku chiku* was significantly predicted by failure (*β* = .41, *t* = 3.66, *p* < .01, *VIF* = 1.00). In summary, the six sadness-pain words were determined to be associated with specific sadness situations.

### Analysis 3: The relation among body parts, pain-expressive properties, and sadness situations for the sadness-pain words

For analysis 3, a hierarchical cluster analysis was first performed for each sadness-pain word in order to group body parts that have similar word-body properties. Consequently, four words (*zuki zuki*, *zukin zukin*, *chiku chiku*, and *shikuri shikuri*) produced five body-part clusters, while the remaining words (*shiku shiku*, *jin jin*) produced four clusters.

Situation-congruent ratings for each word were multiple-regressed, using pain profiles as the independent variable [clustered body-part and pain-expressive properties (continuity, activation, intensity, acuity)] and sex as the control variable. Significant models were found for five words [*zuki zuki* for loneliness: adjusted *R*^*2*^ = .16; *F* (2, 65) = 7.24, *p* < .01; *zukin zukin* for loss: adjusted *R*^*2*^ = .06, *F* (1, 67) = 5.04, *p* < .05; *shiku shiku* for breakup: adjusted *R*^*2*^ = .12, *F* (2, 66) = 5.49, *p* < .01; *chiku chiku* for failure: adjusted *R*^*2*^ = .23, *F* (2, 66) = 10.97, *p* < .01; *shikuri shikuri* for diseases or injury: adjusted *R*^*2*^ = .08, *F* (2, 65) = 3.85, *p* < .05].

**Fig 3:**
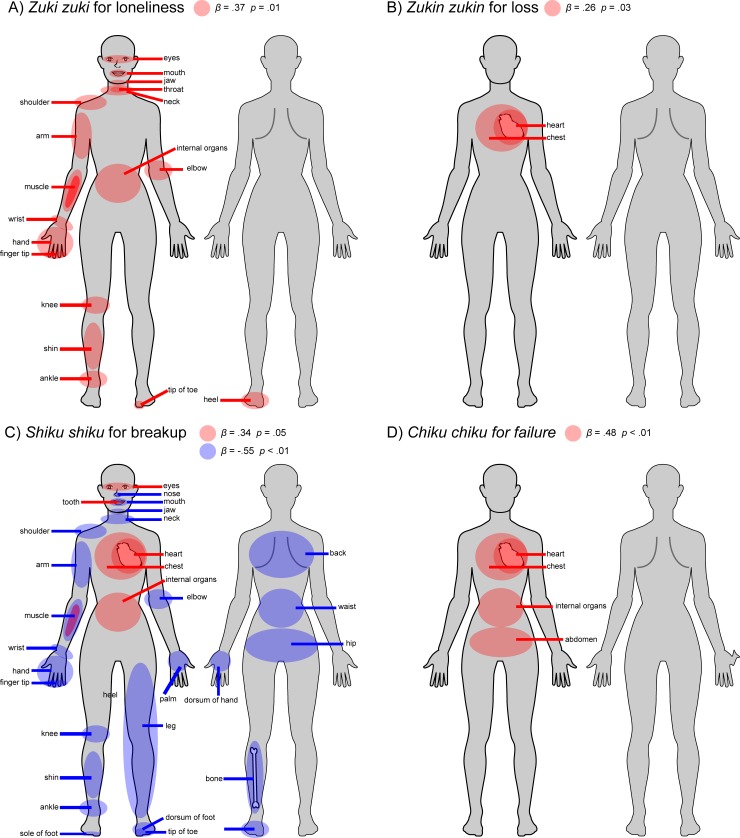
Body maps for each situation-dependent sadness-pain word. In each figure, the color-marked body parts are those that were determined in analysis 3 to be significantly associated with situational-dependent sadness-pain words. A) indicates the 18 body parts that were significant predictors for *zuki zuki* for loneliness, B) indicates the two body parts that were significant predictors for *zukin zukin* for loss, C) indicates the 25 body parts (blue) that were negative predictors and the five parts that were positive predictors for *shiku shiku* for breakup, and D) indicates the four body parts that were significant predictors of *chiku chiku* for failure. A red circle signifies a positive predictor, while a blue circle signifies a negative predictor.

The congruency between *zuki zuki* and loneliness was significantly predicted by body-part cluster 1 (*β* = .37, *t* = 2.56, *p* = .01, *VIF* = 1.04) and by sex (*β* = .29, *t* = 3.27, *p* < .01, *VIF* = 1.04); cluster 1 comprised 18 body parts [head-neck (five): eyes, mouth, jaw, neck, and throat; trunk (one): shoulder; limbs (10): arm, elbow, hand, wrist, ankle, knee, shin, fingertip, tip of toe, and heel; and others (two): muscle and other internal organs] (Fig 3A). Meanwhile, the congruency between *zukin zukin* and loss was significantly predicted by cluster 3 (*β* = .26, *t* = 2.24, *p* = .03, *VIF* = 1.00), which comprised two body parts (chest and heart) (Fig 3B). For the congruency between *shiku shiku* and breakup, cluster 2 and 4 were significant predictors (cluster 2: *β* = −.55, *t* = 3.28, *p* < .01; cluster 4: *β* = .34, *t* = 2.04, *p* = .05, *VIF* = 2.13). Cluster 2 consisted of 25 body parts [head-neck (four): nose, mouth, jaw, and neck; trunk (four): shoulder, waist, back, and hip; limbs (15): arm, elbow, hand, leg, wrist, ankle, knee, shin, fingertip, heel, palm, dorsum of hand, sole of foot, dorsum of foot, and tip of toe; others (two): bone and muscle] and cluster 4 comprised five parts (eyes, tooth, chest, internal organs, and heart) (Fig 3C).

The congruency between *chiku chiku* and failure was significantly predicted by cluster 5 (*β* = .48, *t* = 4.43, *p* < .01, *VIF* = 1.03) and pain intensity (*β* = .25, *t* = 2.32, *p* < .01, *VIF* = 1.03). Cluster 5 consisted of four body parts (stomach, heart, abdomen, and chest) (Fig 3D). For *shikuri shikuri*, cluster 1 and 2 were significant predictors, but high multicollinearity was observed between them (*VIF* = 24.42), both ratings were integrated into one item and re-introduced into the multiple regression analysis; no significant regression model was observed.

## Discussion

The present study aimed to elucidate how psychological foundations support the shared mental representation between sadness and physical pain as a single concept in our long-term memory. For this, we prepared a verbal experimental paradigm including a three-stage research investigation: the first stage concerned identifying sadness-pain words, the second concerned identifying the relations between these sadness-pain words and specific sadness situations, and the third concerned identifying pain-related characteristics (body parts and properties of pain) associated with the sadness-pain words. We determined that some onomatopoeic words represent both sadness and physical pain and are closely related to specific physical and situational properties. First, seven words were identified as being sadness-pain words: *shiku shiku*, *shikuri shikuri*, *zuki zuki*, *gusa gusa*, *chiku chiku*, *zukin zukin*, and *jin jin*. Then, these sadness-pain words were determined to be selectively related to specific sadness situations; for example, the high level of sadness implied by *zukin zukin* is related to loss-situations (funeral). Third, the situation-sensitive sadness-pain words were determined to be associated with specific body parts and pain properties, respectively; for example, the high congruency between *zukin zukin* and loss situations was related to high congruency with specific body parts, such as the chest and heart. Thus, sadness and physical pain can be represented by single verbal concepts based on features related to physical and situational properties.

The identified relation between sadness and physical pain supports the theory that emotional experiences are an interactive product of exteroception and interoception [42], based on the interaction between the body/mind and social situations. Exteroception is defined as sensing stimuli that originate from environments outside the body, while interoception is defined as sensing the internal bodily condition [43]. The interoceptive inference theory [44] posits that emotional experiences are based on both interoceptive and extroceptive changes in physical conditions. Both sadness and pain possess extroceptive aspects, as they are dependent on the perception of external stimuli in specific environments, and they enable us to execute appropriate responses to such situations [4, 27, 45]. Sadness and physical pain are also fundamentally related to interoception occurring within the body. Sensitivity to subtle changes within the body modulates subjective and emotional experiences, including sadness and pain [44, 46], which explains why individuals encountering the same external situations can have differing subjective experiences. The present findings suggest that sadness-pain concepts are represented by memorizing features of not only internal physiological changes, but also information on body parts and the relevant social situations. In this sense, the body is the essential reference site for integrating interoceptive and extroceptive aspects of emotional experiences, yielding the psychological foundation for sadness-pain concepts.

Representing a psychological state by using a sadness-pain concept is advantageous for describing not only self-emotional states, but also cognitive (e.g., why our emotional state is in its current condition) and social information (e.g., how we must act or respond to situations), and also for inferring others’ states and sharing feelings [3]. Such integrated aspects of the sadness-pain concept are also supported clinically. Inadequately referring to and appropriately integrating extroceptive and interoceptive information has been found to evoke emotional dysfunction because identifying specific emotional states has adaptive value [47, 48]. People with depressive symptoms, for example, change the way in which the self is situated in social situations, showing less sensitivity to situational information in their emotional processing [49] and a diminished ability to appropriately differentiate emotional experiences [47]. It can be concluded that topological interaction between the body and mind, and the situation in question is fundamental for constructing emotional concepts that guide us towards taking functional actions in response to the demands of the situation.

Specifically, several such concepts can represent the unique relation between sadness and physical pain profiles. *Zuki zuki* (throbbing, grinding pain) is related to loneliness-sadness and represents throbbing pain, which is generally transmitted by group C nerve fibers and is evoked by both tissue and nerve injuries [50]; consequently, *zuki zuki* should be applicable for expressing pain in a wide range of body parts. Accordingly, the present results show highly congruent associations between *zuki zuki* and numerous body parts; its congruency with loneliness is associated with a congruency with a large cluster of 18 body parts (see Fig 3A). Throbbing pain in specific parts of the body (e.g., the jaw) may not always represent loneliness-sadness. However, when people possess higher sensitivity to the congruency between loneliness-related body parts, they can also more appropriately judge congruency with other body parts, because it is likely that they have the ability to detect slight state changes within their bodies. Additionally, sensitivity to loneliness-sadness is probably important for maintaining a good social life. Social connectedness is crucial for maintaining psychological well-being [51], and, in order to maintain well-being, people must monitor and recognize loneliness as an alarm signal for social rejection or isolation [13, 52]. Therefore, people with high interoception can sensitively recognize the relation between specific pain and certain body parts and use specific pain-related terms to describe loneliness-sadness; this allows them to more easily identify their sadness states and consequently maintain well-being. On the other hand, the control variable of sex also predicted the congruency rating of loneliness-sadness. Female participants showed higher congruency ratings than did male participants (women = 5.67, men = 4.28). Sex-related differences regarding perceiving physical pain have been widely reported; for example, women have been found to possess greater sensitivity to pain stimuli than do men [53]; women have also been found to show greater stress responses to social rejection, evoking loneliness-sadness, than do men [54]. Therefore, women may, more sensitively use pain-related words to represent loneliness-sadness than men would.

*Zukin zukin* (continuous throbbing pain) is another expression for throbbing pain, and is related to loss-situations; specifically, *zukin zukin* represents incessant throbbing pain. Loss-sadness yields a continuous arousal response [16] and, when it is prolonged, often leads to depressive symptoms [55]. The continuous nature of the term provides the psychological basis for the high congruency between *zukin zukin* and loss-sadness. The congruency for loss-sadness was predicted by two body parts, the chest and heart. The expression “heartache” tends to be utilized as a sadness-expressive word [3, 15, 16]; furthermore, interoceptive sensitivity to sadness can be measured by determining how accurately participants can perceive their heartbeat (e.g., [56]); greater increases in interoceptive awareness are associated with stronger emotional experiences (e.g., [57]). Therefore, the present finding regarding *zukin zukin* also confirms that the chest and heart are associated with awareness of interoception, and referencing them can be considered a symbolic expression reflecting a common belief that emotion is grounded in bodily responses [46].

The congruency rating of *shiku shiku* (continuous spasms of dull pain) for breakup-sadness was predicted by two body clusters: 25 body parts and five body parts (see Fig 3C). *Shiku shiku* represents dull pain, which is predominately transmitted by group C nerve fibers that also transmit blunt pressure [50] and radiating pain [58, 59]. The cluster comprising 25 body parts was a negative predictor, indicating that the use of *shiku shiku* for breakup-sadness is not applicable for a wide range of body parts. On the other hand, the five body parts (eyes, tooth, chest, internal organs, and heart) were a positive predictor; people with more sensitive localization of extroceptive stimuli perceived through these body parts are more likely to use *shiku shiku* to describe breakup-sadness feelings. Further, among these body parts the chest and heart were found to be related through *zukin zukin* to loss-sadness, indicating that breakup-sadness shares some properties with loss-sadness. Similar to loss-sadness, sadness feelings relating to a breakup are symbolized by “heartbreak” expressions, which are commonly used in daily life.

The congruency between c*hiku chiku* (repeated pricking pain) and failure was significantly predicted by four body parts: the stomach, heart, abdomen, and chest (Fig 3D). *Chiku chiku*, differing from the above four words for pains associated with group C nerve fiber, is associated with Aδ-fiber [50], and represents a pricking pain such as that evoked by a needle entering the skin [60]; this is qualitatively distinguished from dull or throbbing pain [50]. Such neural-based discrepancy also supports the theory that loss- and failure-sadness possess different features, comprising different subtypes in the major emotional category of sadness [3, 16]. It is likely that internal body parts such as the heart or stomach function as fundamental body parts for integrating extroceptive and interoceptive information, as stressful life events can cause stomach aches [61]. Another predictor of the congruency between *chiku chiku* and failure-sadness is the intensity of the pain. Compared to group C nerve fibers, Aδ-fibers mediate the pain represented by *chiku chiku*, resulting in less engagement in homeostatic and interoceptive functions [50]; at the same intensity of pain, it is likely that Aδ-fiber works less actively than does C-fiber in referring interoceptive information and maintaining homeostasis. However, if the pain level becomes intense, interoceptive information is useful. Therefore, people who experience a more intense pricking pain tend to consider *chiku chiku* to be more applicable to failure-sadness because they can more closely attribute it to their internal states.

The present results have implications for the efficacy of representing mental states in terms of verbal knowledge. Verbally recognizing our own feelings and other’s mental states may contribute to evaluating emotional states to promote mutual communications. Quantitatively identifying emotional states may be often difficult even for ourselves due to subjective ambiguity and variation. As revealed in the present study, on the other hand, specific physical pain words can represent subtypes of sadness. Such metaphorical expressions may promote to identify vague emotional states because intensity of physical pains is relatively easy to assess for ourselves, and can, by extension, represent subtle nuances of emotions such as “Loss of a loved one makes my heart ache.” Furthermore, “affect labeling,” which is putting feelings into words, has been focused on as one of the emotion regulation strategies in research domains [62]. Affect labeling leads to better psychological outcomes because it facilitates the identification of own feelings accurately, which decrease negative emotions [63]. Therefore, sensitively verbalizing various types of sadness via physical pain words may contribute to healing sadness.

In conclusion, sadness and specific physical pain can be represented as unique emotional word. Each sadness-pain word is selectively related to a specific sadness situation and specific body parts. Sadness-pain concepts, therefore, construct knowledge of emotion based on features representing the interaction between the body/mind and external environments, providing advantageous information for adapting to situational demands encountered throughout life.

## Supporting information

S1 Data Data for Analysis 1. Congruency ratings between the sadness-expressive words and the four majour emotions. (XLSX)

S2 Data Data for Analysis 2. Congruency ratings between the sadness-expressive words and the sadness-related situations. (XLSX)

S3 Data Data for Analysis 3. Situation-congruent ratings between the sadness-expressive words and the body parts, characteristics of pain expression. (XLSX)

## References

[1] Duncan G. Mind-body dualism and the biopsychosocial model of pain: what did Descartes really say? J Med Philos. 2000;25:485–513. 10.1076/0360-5310(200008)25:4;1-A;FT485 10916180

[2] Rosch E, Mervis CB, Gray WD, Johnson DM, Boyes-Braem PJCp. Basic objects in natural categories. Cogn Psychol. 1976;8:382–439.

[3] Shirai M, Soshi T, Suzuki N. Knowledge of Sadness: Emotion-related behavioral words differently encode loss and failure sadness. Curr Psychol. 2018;1–15. 10.1007/s12144-018-0010-9

[4] Barrett LF. Solving the emotion paradox: Categorization and the experience of emotion. Pers Soc Psychol Rev. 2006;10:20–46. 10.1207/s15327957pspr1001_2 16430327

[5] Herbert BM, Pollatos O. The body in the mind: on the relationship between interoception and embodiment. Top Cogn Sci. 2012;4:692–704. 10.1111/j.1756-8765.2012.01189.x 22389201

[6] Moors A. Theories of emotion causation: A review. Cogn Emot. 2009;23:625–662.

[7] Plutick R. Emotion: A psychoevolutionary synthesis. New York: Harper & Row; 1980.

[8] Plutick R. Emotions and life: Perspective from psychology, biology, and evolution. Washington, DC: American Psychological Association; 2002.

[9] Shiota MN, Kalat JW. Emotion (2nd ed.). New York: Wadworth; 2011.

[10] Barrett LF. Psychological Construction: The Darwinian Approach to the Science of Emotion. Emot Rev. 2013;5:379–389.

[11] Damasio A, Carvalho GB. The nature of feelings: evolutionary and neurobiological origins. Nat. Rev. Neurosci. 2013;14:143–152. 10.1038/nrn3403 23329161

[12] James W. II.—WHAT IS AN EMOTION? Mind. 1884;9:188–205.

[13] Eisenberger NI, Lieberman MD. Why rejection hurts: a common neural alarm system for physical and social pain. Trends Cogn Sci. 2004;8:294–300. 10.1016/j.tics.2004.05.010 15242688

[14] MacDonald G, Leary MRJP. Why does social exclusion hurt? The relationship between social and physical pain. Psychological bulletin. Psychol Bull. 2005;131:202–223. 10.1037/0033-2909.131.2.202 15740417

[15] Shirai M, Suzuki N. The features of sadness and its time-sequence changes in six types of situations eliciting sadness. Jpn J Res Emot. 2016;23:59–67.

[16] Shirai M, Suzuki N. Is sadness only one emotion? Psychological and physiological responses to sadness induced by two different situations:“loss of someone” and “failure to achieve a goal”. Front Psychol. 2017;8:288 10.3389/fpsyg.2017.00288 28316577 PMC5334320

[17] Frijda NH. The emotions. New York: Press Syndicate of the University of Cambridge; 1986.

[18] Malatesta CZ, Wilson AJBJoSP. Emotion cognition interaction in personality development: A discrete emotions, functionalist analysis. Br J Soc Psychol. 1988;27:91–112. 3370409 10.1111/j.2044-8309.1988.tb00807.x

[19] Lench HC, Tibbett TP, Bench SW. Exploring the toolkit of emotion: What do sadness and anger do for us? Soc Personal Psychol Compass. 2016;10:11–25.

[20] Vingerhoets A. Why only humans weep: Unravelling the mysteries of tears. Oxford: Oxford University Press; 2013.

[21] Baliki MN, Apkarian AV. Nociception, Pain, Negative Moods, and Behavior Selection. Neuron. 2015;87:474–491. 10.1016/j.neuron.2015.06.005 26247858 PMC4529956

[22] Basbaum AI, Bautista DM, Scherrer G, Julius D. Cellular and molecular mechanisms of pain. Cell. 2009;139:267–284. 10.1016/j.cell.2009.09.028 19837031 PMC2852643

[23] Merskey H. The definition of pain. European psychiatry. 1991;6:153–159.

[24] Craig A. A new view of pain as a homeostatic emotion. Trends in Neurosciences. 2003;26:303–307. 12798599 10.1016/s0166-2236(03)00123-1

[25] Craig AD. Emotional moments across time: a possible neural basis for time perception in the anterior insula. Philos Trans R Soc Lond B Biol Sci. 2009;364:1933–1942. 10.1098/rstb.2009.0008 19487195 PMC2685814

[26] Shirai M, Suzuki N. The classification and structures of situations eliciting sadness. Jpn J Res Emot. 2013;20:105–12.

[27] Thornhill R, Thornhill NW. The evolution of psychological pain. Bell R, editor. Lubbock: Texas Tech University Press; 1989.

[28] Eccleston C, Crombez G. Pain demands attention: A cognitive–affective model of the interruptive function of pain. Psychological bulletin. Psychol Bull. 1999;125:356–366. 10349356 10.1037/0033-2909.125.3.356

[29] Berryman C, Stanton TR, Bowering KJ, Tabor A, McFarlane A, Moseley GL. Do people with chronic pain have impaired executive function? A meta-analytical review. Clin Psychol Rev. 2014;34:563–579. 10.1016/j.cpr.2014.08.003 25265056

[30] Grisart JM, Plaghki LH. Impaired selective attention in chronic pain patients. Eur J Pain. 1999;3:325–333. 10.1053/eujp.1999.0138 10700360

[31] Barrett NF, Schulkin J. A. Neurodynamic Perspective on Musical Enjoyment: The Role of Emotional Granularity. Front Psychol. 2017;8:2187 10.3389/fpsyg.2017.02187 29321756 PMC5733545

[32] Barsalou LW, Simmons WK, Barbey AK, Wilson CD. Grounding conceptual knowledge in modality-specific systems. Trends Cogn Sci. 2003;7:84–91. 12584027 10.1016/s1364-6613(02)00029-3

[33] Inose H. Translating Japanese onomatopoeia and mimetic words. Translation Research Projects. 2007 1:97–116.

[34] Tamori I. Onomatope: Giongo/Gitaigo wo tanoshimu [Onomatopoeia: enjoy giongo/gitaigo]. Tokyo: Iwanami; 2002.

[35] Imai M, Kita S, Nagumo M, Okada H. Sound symbolism facilitates early verb learning. Cognition. 2008;109:54–65. 10.1016/j.cognition.2008.07.015 18835600

[36] Ozturk O, Krehm M, Vouloumanos A. Sound symbolism in infancy: evidence for sound–shape cross-modal correspondences in 4-month-olds. J Exp Child Psychol. 2013;114:173–186. 10.1016/j.jecp.2012.05.004 22960203

[37] Ono M. Giongo/Gitaigo 4500 nihongo onomatope jiten [Japanese Onomatopoeia Dictionary 4500 mimetics]. Tokyo: Shogakukan; 2007.

[38] Kelman L. Pain characteristics of the acute migraine attack. Headache: J Headache Pain. 2006;46:942–953. 10.1111/j.1526-4610.2006.00443.x 16732840

[39] Watson KD, Papageorgiou AC, Jones GT, Taylor S, Symmons DP, Silman AJ, et al Low back pain in schoolchildren: occurrence and characteristics. Pain. 2002:87–92. 12031782 10.1016/s0304-3959(02)00008-8

[40] Wirth B, Humphreys BK. Pain characteristics of adolescent spinal pain. BMC Pediatr. 2015;15:42 10.1186/s12887-015-0344-5 25886130 PMC4411747

[41] Ekman P. An argument for basic emotions. Cogn Emot. 1992;6:169–200.

[42] Filippetti ML, Tsakiris M. Heartfelt embodiment: Changes in body-ownership and self-identification produce distinct changes in interoceptive accuracy. Cognition. 2017;159:1–10. 10.1016/j.cognition.2016.11.002 27880880

[43] Craig AD. How do you feel? Interoception: the sense of the physiological condition of the body. Nat Rev Neurosci. 2002;3:655–666. 10.1038/nrn894 12154366

[44] Seth AK. Interoceptive inference, emotion, and the embodied self. Trends Cogn Sci. 2013;17:565–573. 10.1016/j.tics.2013.09.007 24126130

[45] Nesse RM. Evolutionary explanations of emotions. Hum Nat. 1990;1:261–289. 10.1007/BF02733986 24222085

[46] Dunn BD, Galton HC, Morgan R, Evans D, Oliver C, Meyer M, et al Listening to your heart. How interoception shapes emotion experience and intuitive decision making. Psychol Sci. 2010;21:1835–44. 10.1177/0956797610389191 21106893

[47] Demiralp E, Thompson R, Mata J, Jaeggi S, Buschkuehl M, Barrett L, et al Feeling Blue or Turquoise? Emotional Differentiation in Major Depressive Disorder. Psychol Sci. 2012;23:1–7. 10.1177/0956797612444903 PMC4004625 23070307

[48] Schwarz N, Clore GL. Feelings and phenomenal experiences. 2 ed New York: Guilford; 2007.

[49] Rottenberg J, Gross JJ, Gotlib IH. Emotion context insensitivity in major depressive disorder. J Abnorm Psychol. 2005;114:627–639. 10.1037/0021-843X.114.4.627 16351385

[50] Beissner F, Brandau A, Henke C, Felden L, Baumgartner U, Treede RD, et al Quick discrimination of A(delta) and C fiber mediated pain based on three verbal descriptors. PLoS One. 2010;5:e12944 10.1371/journal.pone.0012944 20886070 PMC2944851

[51] Leary MR, Tambor ES, Terdal SK, Downs DL. Self-esteem as an interpersonal monitor: The sociometer hypothesis. J Pers Soc Psychol. 1995;68:518–530.

[52] Panksepp J, Normansell L, Herman B, Bishop P, Crepeau L. Neural and neurochemical control of the separation distress call The physiological control of mammalian vocalization: Springer; 1988 p. 263–99.

[53] Fillingim RB. Sex, gender, and pain: women and men really are different. Curr Rev Pain. 2000;4:24–30. 10998712 10.1007/s11916-000-0006-6

[54] Stroud LR, Salovey P, Epel ES. Sex differences in stress responses: social rejection versus achievement stress. Biol Psychiatry. 2002;52:318–327. 12208639 10.1016/s0006-3223(02)01333-1

[55] Sander D, Scherer KR. The Oxford companion to emotion and the affective sciences. Oxford: Oxford University Press; 2009.

[56] Critchley HD, Wiens S, Rotshtein P, Öhman A, Dolan R. Neural systems supporting interoceptive awareness. Nat Neurosci. 2004;7:189–195. 10.1038/nn1176 14730305

[57] Barrett LF, Quigley KS, Bliss-Moreau E, Aronson KR. Interoceptive sensitivity and self-reports of emotional experience. J Pers Soc Psychol. 2004;87:684–697. 10.1037/0022-3514.87.5.684 15535779 PMC1224728

[58] Mumford J, Bowsher DJP. Pain and protopathic sensibility. A review with particular reference to the teeth. Pain. 1976;2:223–243. 800250 10.1016/0304-3959(76)90002-6

[59] Sessle B, Hu J, Amano N, Zhong G. Convergence of cutaneous, tooth pulp, visceral, neck and muscle afferents onto nociceptive and non-nociceptive neurones in trigeminal subnucleus caudalis (medullary dorsal horn) and its implications for referred pain. Pain. 1986;27:219–35. 3797017 10.1016/0304-3959(86)90213-7

[60] Bowsher D. Neurogenic pain syndromes and their management. Br Med Bull. 1991;47:644–666. 1794077 10.1093/oxfordjournals.bmb.a072498

[61] Gibbs Jr RW. Embodied experience and linguistic meaning. Brain Lang. 2003;84:1–15. 12537948 10.1016/s0093-934x(02)00517-5

[62] Torre JB, Lieberman MD. Putting Feelings Into Words: Affect Labeling as Implicit Emotion Regulation. Emot Rev. 2018;10:116–124.

[63] Mendolia M, Kleck RE. Effects of talking about a stressful event on arousal: Does what we talk about make a difference? J Pers Soc Psychol. 1993;64: 283–292. 8433274 10.1037//0022-3514.64.2.283

